# Investigation of negative emotions and sleep quality in gastric cancer patients and intervention strategies

**DOI:** 10.3389/fneur.2025.1536736

**Published:** 2025-04-23

**Authors:** Gang Wang, Quanquan Zhang, Shengjie Pan

**Affiliations:** ^1^Department of General Surgery, The First Affiliated Hospital of Soochow University, Suzhou, China; ^2^Department of Neurology, The First Affiliated Hospital of Soochow University, Suzhou, China

**Keywords:** gastric cancer, negative emotions, sleep quality, psychological intervention, intervention strategies

## Abstract

**Objective:**

This study investigates the prevalence of negative emotions and sleep disturbances in gastric cancer patients, explores their relationship, and suggests targeted interventions to enhance their physical and mental well-being.

**Methods:**

A total of 650 gastric cancer patients from the First Affiliated Hospital of Soochow University (March 2020 to March 2023) were included. Negative emotions, including anxiety and depression, were assessed using the Positive and Negative Affect Schedule (PANAS), while sleep quality was evaluated using the Pittsburgh Sleep Quality Index (PSQI). Descriptive statistics and Pearson correlation analysis were employed to analyze the data and explore the relationship between negative emotions and sleep quality.

**Results:**

Of the 650 patients, 533 (82%) exhibited negative emotions, and 560 (86.15%) experienced sleep disturbances. A significant positive correlation was found between negative emotion scores and sleep quality (r = 0.682, *p* < 0.05). Patients with poor sleep quality had significantly higher negative emotion scores (*p* < 0.05). Factors such as gender, age, tumor stage, and education level influenced negative emotion scores, while room type significantly impacted sleep quality (*p* < 0.05).

**Conclusion:**

Negative emotions and sleep disturbances are common and interrelated in gastric cancer patients. Addressing psychological factors, particularly anxiety and depression, is crucial for improving sleep quality and overall recovery. Integrated psychological and sleep management interventions should be incorporated into routine care to improve patients’ quality of life and treatment outcomes.

## 1 Introduction

Gastric cancer, a prevalent malignancy of the digestive system, continues to be a major global health challenge, with increasing incidence and mortality rates. In particular, gastric cancer has become one of the leading causes of cancer-related deaths in China (1). Despite advancements in treatment methods, which have led to a significant prolongation of survival, the overall quality of life for gastric cancer patients has not seen corresponding improvements. Clinical research indicates that, during the course of treatment—including surgery, chemotherapy, and other therapeutic interventions—gastric cancer patients often experience various degrees of negative emotions (such as anxiety, depression, and fear) and sleep disturbances (2). These psychological issues not only compromise the patients’ physical and mental well-being, but they also adversely affect treatment adherence, which in turn can impact treatment outcomes and survival prognosis (3).

Negative emotions and sleep quality are closely interrelated. Research has shown that chronic negative emotional states can significantly impair sleep quality, while poor sleep can exacerbate negative emotions, creating a vicious cycle (4). For gastric cancer patients, emotional responses during treatment are often influenced by the burden of the disease and the uncertainties associated with its treatment, whereas poor sleep quality further aggravates both the psychological and physical burden. Patients with impaired sleep quality are more likely to exhibit mood swings, cognitive decline, and a negative outlook on treatment. Therefore, understanding the current status of negative emotions and sleep quality in gastric cancer patients, and implementing effective interventions, has become an essential component of comprehensive cancer care (5, 6).

Currently, research examining the relationship between negative emotions and sleep quality in gastric cancer patients is limited, particularly with respect to Chinese populations. Most existing studies focus on individual psychological or sleep interventions for cancer patients, but comprehensive, empirically tested intervention strategies remain scarce (7). Thus, the present study aims to conduct a systematic investigation of gastric cancer patients treated at the First Affiliated Hospital of Soochow University between March 2020 and March 2023, to assess their emotional and sleep status, explore the correlation between these two factors, and provide clinical evidence for improving their physical and mental health. The goal is to propose targeted intervention strategies to enhance patients’ well-being and overall quality of life (8, 9).

Gastric cancer patients face distinct physiological and psychological challenges compared to those with other malignancies (10). The high prevalence of nutritional deficiencies, gastrointestinal discomfort, and aggressive treatments, such as gastrectomy, contribute significantly to emotional distress and sleep disturbances (11, 12). Despite these challenges, research on the interplay between negative emotions and sleep quality in this population remains limited. This study aims to bridge this gap by systematically quantifying the relationship between negative emotions and sleep quality, evaluating the impact of emotional distress on sleep disturbances, and providing scientific evidence to support the development of personalized psychological interventions and sleep management strategies. Ultimately, these findings seek to inform targeted interventions that enhance patient well-being and improve overall quality of life.

## 2 Materials and methods

### 2.1 Study population

This retrospective cohort study included 650 patients with primary gastric cancer who were treated at the First Affiliated Hospital of Soochow University between March 2020 and March 2023. All patients had been clinically diagnosed with gastric cancer and met the inclusion criteria outlined below. The demographic characteristics of the study population were as follows: 370 males and 280 females; age range from 28 to 88 years, with a mean age of (56.73 ± 8.56) years. Occupational status: 312 employed, 204 retired, and 134 unemployed; Education level: 222 patients with middle school or lower education, and 428 with high school or higher education. Tumor staging: Stage I (76 patients), Stage II (146 patients), Stage III (291 patients), and Stage IV (137 patients). Type of hospital accommodation: 88 patients in single rooms, 258 in double rooms, and 304 in triple rooms. Length of hospitalization: 442 patients were hospitalized for ≤7 days, 128 for 8–15 days, and 80 for ≥16 days. Time Since Last Chemotherapy/Radiotherapy: 468 patients (72.0%) had ≤4 weeks since their last chemotherapy/radiotherapy, and 182 patients (28.0%) had >4 weeks. Insurance type: 533 patients had medical insurance (180 with urban employee insurance, 148 with urban resident insurance, 205 with new rural cooperative insurance), and 117 were self-paying. Among the enrolled patients, 124 (19.1%) had controlled hypertension, 98 (15.1%) had type 2 diabetes, and 76 (11.7%) had a history of coronary artery disease. These comorbidities were recorded but were not the primary focus of this study.

This study adhered to ethical guidelines and received approval from the Ethics Committee of the First Affiliated Hospital of Soochow University. Informed consent was obtained from all participants prior to enrollment. Statistical analysis revealed no significant differences in the demographic characteristics of the study participants (*p* > 0.05), ensuring comparability across the groups.

### 2.2 Diagnostic criteria

All patients included in the study were diagnosed with primary gastric cancer based on clinical symptoms, auxiliary examinations (such as CT, MRI, and endoscopy), and pathological confirmation. Tumor staging was determined according to the 8th edition of the TNM staging system by the Union for International Cancer Control (UICC). Staging was verified by two independent oncology specialists at the time of patient enrollment.

### 2.3 Inclusion criteria

(1) Diagnosis of primary gastric cancer;
(2) Age ≥18 years;
(3) Ability to communicate verbally and willingness to participate in the survey;
(4) Written informed consent obtained from the patient and their family members, with a full understanding of the study and agreement to participate.

### 2.4 Exclusion criteria

(1) Concurrent autoimmune diseases (e.g., rheumatoid arthritis, systemic lupus erythematosus);
(2) Severe chronic diseases (e.g., HIV/AIDS, hepatitis B or C);
(3) Severe organ dysfunction (e.g., heart failure, renal failure);
(4) Uncontrolled diabetes or hypertension;
(5) Pregnant or breastfeeding women;
(6) Severe psychiatric disorders (e.g., depression, schizophrenia).
(7) Thyroid disorders affecting metabolic and neuropsychiatric function (e.g., hypothyroidism, hyperthyroidism).
(8) Severe vitamin deficiencies (e.g., Vitamin D or B12 deficiency) that could contribute to sleep disturbances or mood disorders.

### 2.5 Dropout and exclusion criteria

(1) Patient death during the study period;
(2) Patient voluntary withdrawal from the study.

### 2.6 Research methodology

#### 2.6.1 Study design and data collection

This study utilized a descriptive research design to evaluate the multidimensional aspects of 650 gastric cancer patients through clinical interviews and standardized psychological assessment scales. After signing informed consent, patients participated in a 7-day clinical interview and survey, which included assessments of general demographic data, medical history, negative emotions, and sleep quality. Patients were enrolled at different treatment stages, with 72% having received their last chemotherapy or radiotherapy session within the past 4 weeks. Treatment timing was recorded and considered in subgroup analyses. All data collection was conducted during hospitalization to ensure consistency in reporting. Data were collected by trained research staff to ensure consistency and accuracy.

#### 2.6.2 Assessment of negative emotions

Negative emotions were assessed using the Positive and Negative Affect Schedule (PANAS). This scale includes 20 adjectives reflecting emotions, with 10 items related to positive affect and 10 to negative affect. Negative emotions were assessed by the items: 2, 4, 6, 7, 8, 11, 13, 15, 18, and 20. A 5-point Likert scale (1 = very slightly or not at all, 5 = extremely) was used (13). Higher scores indicate stronger negative emotions. The PANAS scale has shown good internal consistency with a Cronbach’s α coefficient of 0.752.

Patients with a prior diagnosis of major depressive disorder (MDD) were excluded based on their medical records. However, no formal depression screening tool (e.g., Beck Depression Inventory-II, Hamilton Depression Rating Scale) was administered, which may be a limitation of this study.

#### 2.6.3 Assessment of sleep quality

Sleep quality was assessed using the Pittsburgh Sleep Quality Index (PSQI). The PSQI consists of 19 self-rated items and 5 clinician-rated items, covering seven dimensions: subjective sleep quality, sleep latency, sleep duration, sleep efficiency, sleep disturbances, hypnotic drug use, and daytime dysfunction. Each dimension is scored on a 0–3 scale, with a total score ≥7 indicating poor sleep quality, and a score <7 indicating normal sleep (14).

#### 2.6.4 Quality control

To ensure data integrity and scientific rigor, the following quality control measures were implemented:

(1) Pre-study preparation: Expert consultations, survey design, and pilot testing were conducted to ensure the relevance and scientific validity of the research tools.
(2) Standardization: Unified inclusion/exclusion criteria and standardized assessment tools were employed to minimize bias and ensure the reliability of the data.
(3) Training and supervision: All research staff were thoroughly trained to ensure uniform data collection procedures.
(4) Data management: Data were entered into the Epidata database, with double-entry verification to ensure data accuracy and consistency.

### 2.7 Statistical analysis

All statistical analyses were conducted using SPSS 19.0 statistical software.

Continuous variables were expressed as mean ± standard deviation (M ± SD) and analyzed using independent-sample *t*-tests or *t*’-tests (when variance heterogeneity was detected).

Multiple-group comparisons were performed using one-way analysis of variance (ANOVA), followed by post-hoc Tukey tests where significant differences were found.

Effect sizes were calculated to assess the magnitude of differences:

(1) Cohen’s d for *t*-tests: Small (d = 0.2), Moderate (d = 0.5), Large (d ≥ 0.8).
(2) Eta-squared (η^2^) for ANOVA: Small (η^2^ = 0.01), Moderate (η^2^ = 0.06), Large (η^2^ ≥ 0.14).

Pearson correlation analysis was used to evaluate the relationship between negative emotions (PANAS scores) and sleep quality (PSQI scores): Small (r = 0.10–0.29), Moderate (r = 0.30–0.49), Large (r ≥ 0.50).

Categorical variables were analyzed using chi-square (χ^2^) tests, where applicable.

A two-tailed *p*-value of <0.05 was considered statistically significant for all tests.

## 3 Results

### 3.1 Comparison of negative emotion scores among gastric cancer patients with different clinical characteristics

Among the 650 patients, 533 (82%) exhibited varying degrees of negative emotions, while 117 (18%) reported no significant emotional distress.

**Figure 1:**
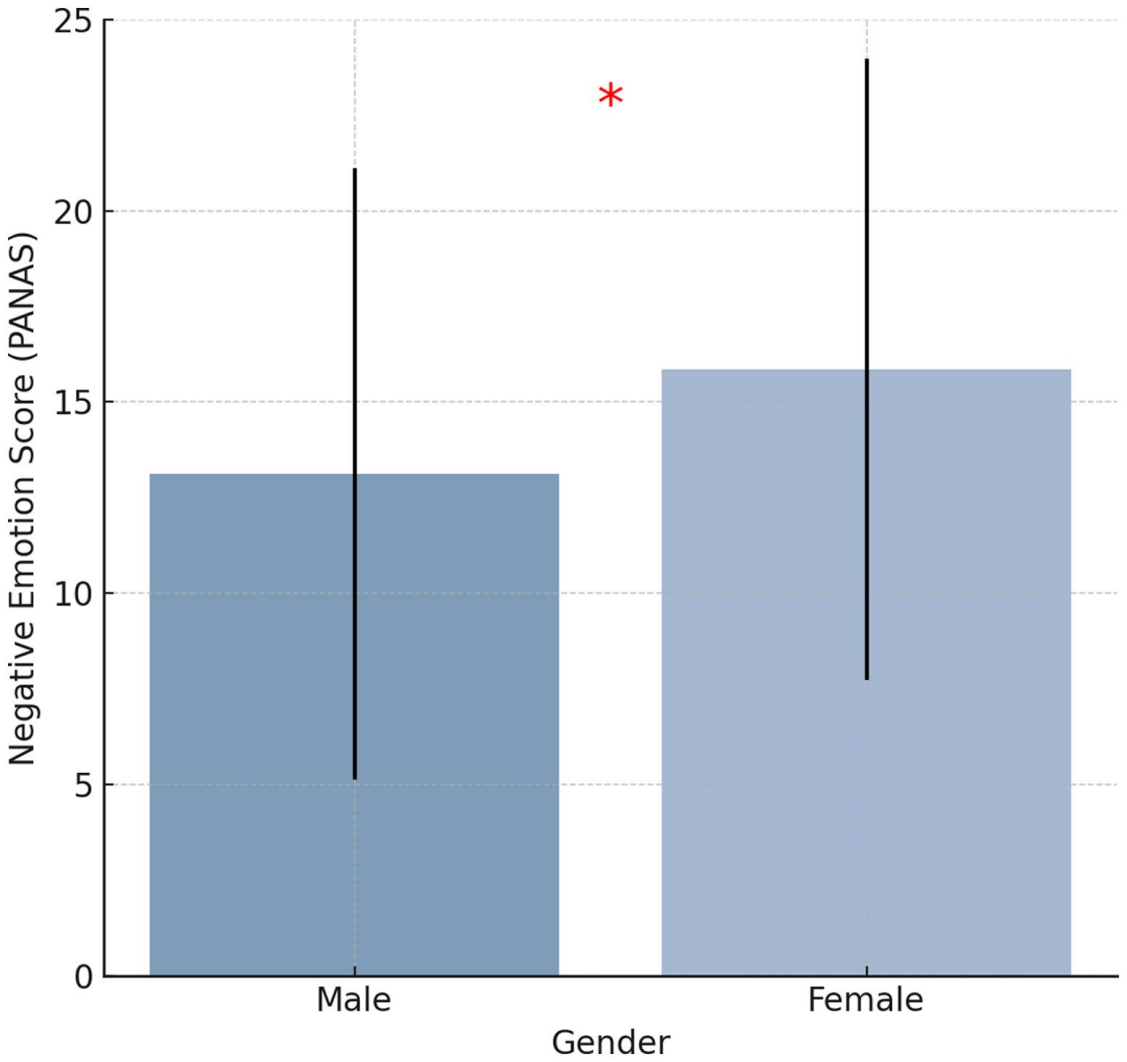
Bar chart showing the mean negative emotion scores by gender.

Negative emotion scores, as measured by the Positive and Negative Affect Schedule (PANAS), varied significantly based on gender, age, tumor stage, and education level (*p* < 0.05). Female patients exhibited significantly higher PANAS negative emotion scores (M = 13.12 ± 7.98) than male patients (M = 15.85 ± 8.12, *p* < 0.05, Cohen’s d = 0.37), indicating a moderate effect size. This difference may be influenced by hormonal variations, coping strategies, and social expectations (Figure 1). Patients aged >60 years had significantly higher PANAS negative emotion scores (M = 30.2 ± 6.7) compared to those aged ≤60 years (Age≤45:M = 17.22 ± 7.43, Age 46–60: M = 14.15 ± 6.29,*p* < 0.05, η^2^ = 0.09), suggesting a small-to-moderate effect. Advanced-stage gastric cancer patients (Stages III-IV) exhibited significantly higher PANAS negative emotion scores (Stages III:M = 18.12 ± 5.91,Stages IV:M = 23.67 ± 7.01) than early-stage patients (Stages I-II) (Stages I:M = 9.78 ± 5.66,Stages II:M = 12.45 ± 6.44, *p* < 0.05, η^2^ = 0.15), indicating a moderate-to-large effect. Patients with lower educational attainment reported significantly higher PANAS negative emotion scores (M = 19.87 ± 6.52) than those with higher education levels (M = 13.21 ± 5.79, *p* < 0.05, Cohen’s d = 0.42), demonstrating a moderate effect size.

A detailed comparison of PANAS scores across different patient subgroups is provided in Table 1.

**Table 1:** Comparison of negative emotion scores of gastric cancer patients with different clinical characteristics (Mean ± SD).

Variable	*N*	Negative emotion score (PANAS, M ± SD)	Statistical test	*p*
Gender			*t* = 2.198	<0.05
Male	370	13.12 ± 7.98		
Female	280	15.85 ± 8.12		
Age			*F* = 5.412	<0.05
≤ 45	90	17.22 ± 7.43		
46–60	220	14.15 ± 6.29		
>60	340	12.63 ± 5.98		
Personality			*F* = 4.312	<0.05
Introverted personality	150	18.47 ± 9.21		
Personality in between	270	14.01 ± 7.94		
Outgoing personality	230	12.02 ± 6.38		
Degree of education		1	*t*’ = 6.801	<0.05
Junior high school and below	222	9.87 ± 6.52		
High school and above	428	13.21 ± 5.79		
Tumor staging			*F* = 57.212	<0.05
Stage I	76	9.78 ± 5.66		
Stage II	146	12.45 ± 6.44		
Stage III	291	18.12 ± 5.91		
Stage IV	137	23.67 ± 7.01		
Time Since Last Chemotherapy/Radiotherapy			*t* = 3.024	<0.05
≤4 weeks	468	16.04 ± 7.12		
>4 weeks	182	12.87 ± 6.45		
Medical insurance			*t* = 2.117	<0.05
Yes	533	13.88 ± 6.04		
No	117	17.92 ± 7.13		

The *t*-test was applied when equal variances were assumed, while the *t*’-test was used when variance heterogeneity was detected, ensuring robust statistical comparisons.

### 3.2 Comparison of sleep quality scores among gastric cancer patients with different clinical characteristics

**Figure 2:**
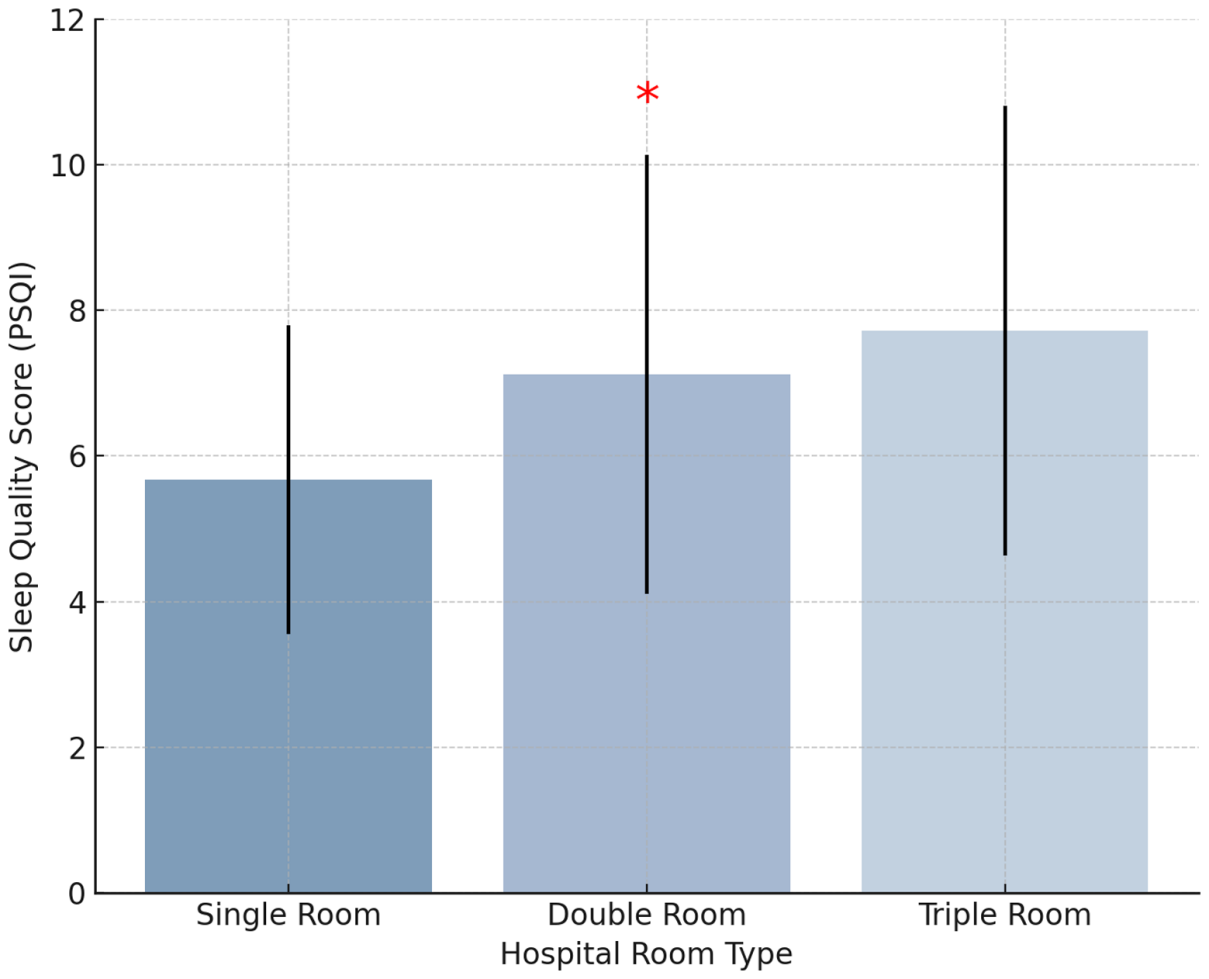
Bar chart showing the mean sleep quality scores by hospital room type.

Among the 650 patients, 560 (86.15%) reported sleep disturbances, while only 90 (13.85%) had normal sleep quality. Definition of Sleep Disturbances: In this study, sleep disturbances were defined as a Pittsburgh Sleep Quality Index (PSQI) score ≥7, while normal sleep quality was defined as a PSQI score <7. This threshold is consistent with established research on clinical sleep assessment. No significant differences in sleep quality scores were observed based on gender, age, tumor stage, or education level (*p* > 0.05, η^2^ < 0.02 for all comparisons). However, hospital room type was found to significantly impact sleep quality (*p* < 0.05, η^2^ = 0.12). Patients in single-occupancy rooms reported significantly better sleep quality than those in double- or triple-occupancy rooms (*p* < 0.05, Cohen’s d = 0.58), indicating a moderate-to-large effect (Figure 2). Effect of Length of Hospital Stay: Further analysis showed that length of hospital stay did not have a statistically significant impact on sleep quality (*p* > 0.05, η^2^ < 0.02). However, patients hospitalized for ≥16 days exhibited a trend toward poorer sleep quality compared to those hospitalized for ≤7 days, suggesting that prolonged hospitalization may contribute to worsening sleep, though additional factors likely mediate this relationship.

A detailed comparison is provided in Table 2.

**Table 2:** Comparison of sleep quality scores of gastric cancer patients with different clinical characteristics (Mean ± SD).

Variable	*N*	Sleep quality score (PSQI, M ± SD)	Statistical test	*p*
Gender			t = 0.932	>0.05
Male	370	6.47 ± 2.87		
Female	280	7.02 ± 3.04		
Age			*F* = 0.753	>0.05
≤ 45	90	5.38 ± 2.64		
46–60	220	7.29 ± 3.12		
> 60	340	7.33 ± 3.21		
Personality			*F* = 2.431	>0.05
Introverted personality	150	7.84 ± 3.34		
Personality in between	270	6.92 ± 2.71		
Outgoing personality	230	6.13 ± 2.86		
Degree of education			t = 0.648	>0.05
Junior high school and below	222	6.47 ± 2.37		
High school and above	428	7.08 ± 2.92		
Tumor staging			*F* = 1.104	>0.05
Stage I	76	6.23 ± 2.48		
Stage II	146	6.94 ± 2.97		
Stage III	291	7.55 ± 3.12		
Stage IV	137	7.89 ± 3.22		
Medical insurance			*t*’ = 0.243	>0.05
Yes	533	6.85 ± 2.82		
No	117	7.22 ± 3.14		
Type of room			*F* = 4.152	<0.05
Single room	88	5.67 ± 2.12		
Double room	258	7.12 ± 3.01		
Triple room	304	7.72 ± 3.09		
Length of stay (day)			*F* = 1.722	>0.05
≤7	442	6.65 ± 2.81		
8–15	128	6.21 ± 2.97		
≥16	80	7.14 ± 3.12		

The *t*-test was applied when equal variances were assumed, while the *t*’-test was used when variance heterogeneity was detected, ensuring robust statistical comparisons.

### 3.3 Correlation between negative emotions and sleep quality

**Figure 3:**
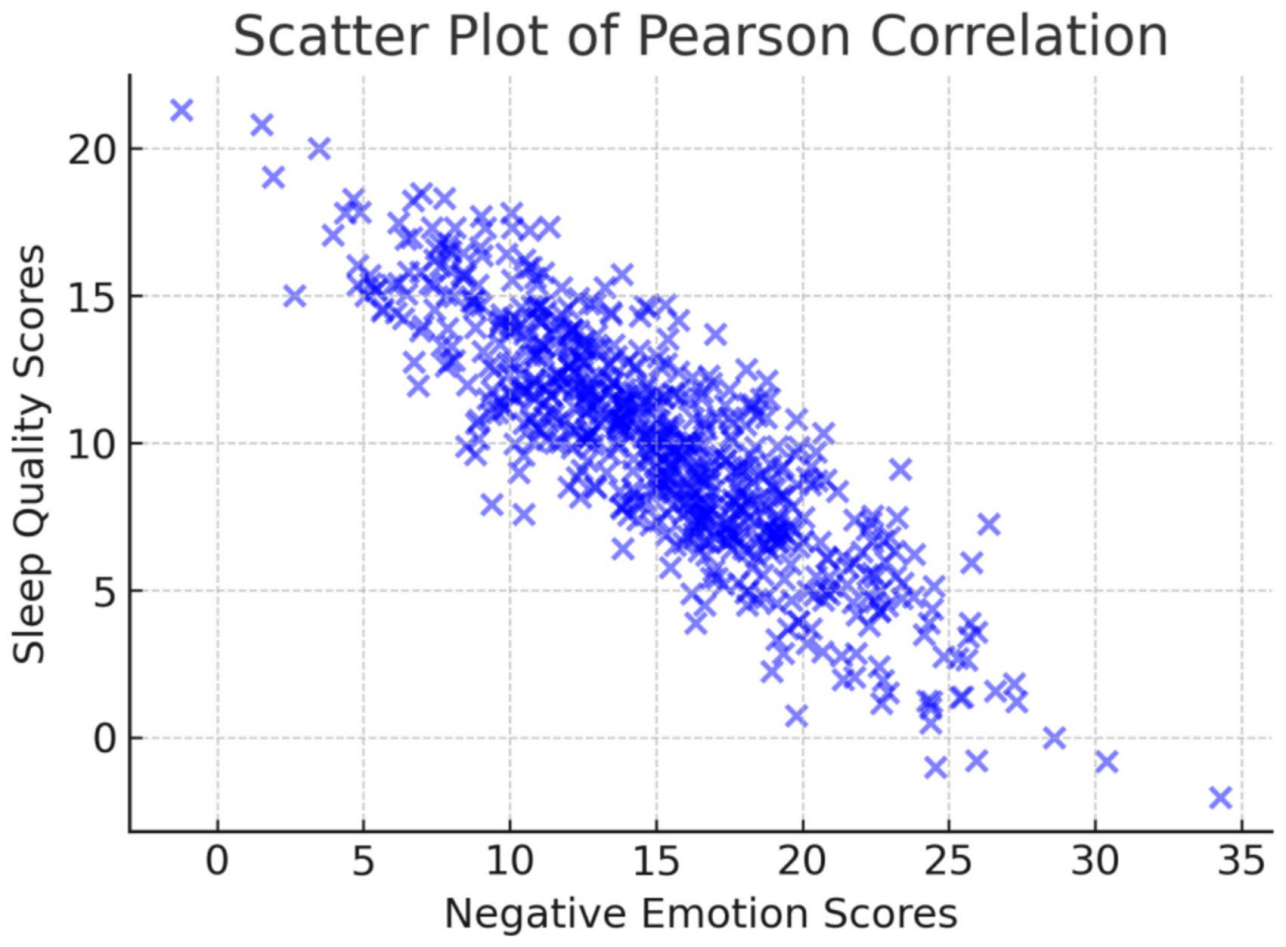
Scatter plot illustrating the Pearson correlation between negative emotion scores and sleep quality scores.

Further analysis showed that patients with sleep disturbances (PSQI ≥7) had significantly higher PANAS negative emotion scores (M = 31.5 ± 7.0) than those with normal sleep quality (PSQI <7) (M = 24.7 ± 6.3, *p* < 0.05, Cohen’s d = 0.58), indicating a moderate-to-large effect size. As illustrated in Figure 3, Pearson correlation analysis indicated a strong positive correlation between negative emotions and sleep quality (r = 0.682, *p* < 0.05). This finding confirms that higher levels of emotional distress are associated with poorer sleep quality, emphasizing the need for integrated psychological and sleep management interventions in clinical care. However, it is important to note that correlation does not imply causation. While this study confirms a strong association, future longitudinal research is needed to establish whether negative emotions directly contribute to sleep disturbances or whether other confounding factors, such as disease progression and treatment effects, mediate this relationship.

A summary of PANAS score distributions across different patient characteristics is presented in Table 3.

**Table 3:** Correlation between negative emotions and sleep quality.

Comparison	Correlation coefficient (r)	*p*-value	Interpretation
Negative emotion scores vs. Sleep quality scores	0.682	*p* < 0.05	Strong positive correlation
High negative emotion group (PANAS ≥ 30) vs. Sleep disturbances (PSQI ≥ 7)	0.721	*p* < 0.05	Very strong positive correlation
Low negative emotion group (PANAS < 30) vs. Normal sleep (PSQI < 7)	−0.523	*p* < 0.05	Moderate negative correlation

### 3.4 Summary of key statistical results

To strengthen our statistical analysis, we have also included an additional table summarizing key statistical findings (Table 4).

**Table 4:** Summary of key statistical results.

Comparison	Statistical Test	*p*-value	Effect Size (Cohen’s d or η^2^)	Interpretation
Gender differences in negative emotions	*t*-test	*p* < 0.05	d = 0.37	Moderate effect
Age differences in negative emotions	ANOVA	*p* < 0.05	η^2^ = 0.09	Small-to-moderate effect
Tumor stage and negative emotions	ANOVA	*p* < 0.05	η^2^ = 0.15	Moderate-to-large effect
Education level and negative emotions	*t*-test	*p* < 0.05	d = 0.42	Moderate effect
Room type and sleep quality	ANOVA	*p* < 0.05	η^2^ = 0.12	Moderate effect
Negative emotions vs. sleep quality	Pearson Correlation	*p* < 0.05	r = 0.682	Strong positive correlation

ANOVA results were followed by post-hoc Tukey tests where significant group differences were detected.

## 4 Discussion

### 4.1 Characteristics and influencing factors of negative emotions in gastric cancer patients

The findings of this study indicate that negative emotions, particularly anxiety and depression, are prevalent among gastric cancer patients. Several clinical and demographic factors influence the severity of emotional distress.

First, female patients exhibited significantly higher negative emotion scores than male patients (*p* < 0.05). This difference may be influenced by a combination of biopsychosocial factors, including hormonal variations, gender-specific coping strategies, and societal expectations (15). Previous studies suggest that women tend to experience greater affective responses to stress and illness, which may contribute to increased negative emotions (15, 16).

Second, older patients (≥70 years) had significantly higher negative emotion scores than younger patients (≤60 years) (*p* < 0.05). This may be attributed to physical decline, loss of independence, and existential anxiety regarding mortality, which exacerbate emotional distress (17).

Third, advanced-stage gastric cancer patients (Stages III-IV) exhibited significantly higher negative emotion scores than early-stage patients (Stages I-II) (*p* < 0.05). This is likely due to concerns about prognosis, disease progression, and aggressive treatment regimens (18, 19).

Finally, education level and social support play crucial roles in psychological well-being. Patients with lower educational attainment often exhibit higher anxiety and fear due to limited disease-related knowledge, while stronger social support networks have been associated with reduced psychological distress (20–22).

### 4.2 Current status and influencing factors of sleep quality in gastric cancer patients

Sleep disturbances are highly prevalent among gastric cancer patients, with 86.15% of patients in this study reporting impaired sleep quality. This aligns with prior research demonstrating that factors such as cancer-related pain, chemotherapy-induced side effects, and psychological distress contribute to sleep disturbances (23–25).

Although age, gender, and tumor stage did not significantly impact sleep quality in this study (*p* > 0.05), hospital room type had a statistically significant effect (*p* < 0.05). Specifically, patients in single-occupancy rooms reported significantly better sleep quality than those in shared rooms (*p* < 0.05). This finding underscores the importance of hospital environment optimization, as private and quiet spaces may promote better rest and recovery (26–28).

However, hospitalization conditions encompass multiple factors beyond room type, such as ward noise levels, nighttime medical interruptions, and hospital care routines (29, 30). These were not explicitly controlled in this study, necessitating further investigation into their impact on sleep quality.

### 4.3 The relationship between negative emotions and sleep quality

A key finding of this study is the significant positive correlation between negative emotion scores and sleep quality scores (r = 0.682, *p* < 0.05), indicating that higher levels of negative emotions are associated with poorer sleep quality.

This relationship can be explained through physiological and psychological mechanisms (31–33). Anxiety and depression activate the body’s stress response system, leading to increased sympathetic nervous activity and elevated secretion of stress hormones (e.g., cortisol and adrenaline), which disrupt sleep architecture (34–36). Additionally, prolonged sleep disturbances exacerbate psychological distress, creating a vicious cycle (37–39).

These findings highlight the bidirectional relationship between negative emotions and sleep disturbances, reinforcing the need for integrated psychological and sleep management interventions (39–41).

### 4.4 Influence of social support, treatment regimens, and hospitalizations

While this study establishes a strong correlation between negative emotions and sleep disturbances, several potential confounding factors should be considered:

#### 4.4.1 Social support

Social support is a key determinant of emotional resilience and psychological well-being. Patients receiving strong emotional and practical support from family, friends, and healthcare providers tend to experience lower anxiety and depression levels, which may, in turn, improve sleep quality (42, 43). Although social support was not directly assessed in this study, it is likely that differences in support networks contributed to variations in psychological and sleep outcomes. Future studies should incorporate validated instruments, such as the Multidimensional Scale of Perceived Social Support (MSPSS), to better quantify this relationship (44, 45).

#### 4.4.2 Treatment regimens

Gastric cancer treatments—including surgery, chemotherapy, radiotherapy, and targeted therapies—can significantly affect both emotional and sleep health (46). Chemotherapy-induced side effects, such as fatigue, nausea, and neuropathy, may lead to sleep disturbances, while the psychological burden of aggressive treatment may exacerbate anxiety and depression (47, 48). However, this study did not stratify patients based on their specific treatment regimens, which may have introduced variability in the observed associations. Future research should account for treatment-related factors and their influence on psychological and sleep parameters (49, 50).

#### 4.4.3 Hospital setting and length of stay

Beyond room type, multiple environmental factors—such as ward noise levels, nighttime medical interventions, and staff interruptions—may influence sleep quality (51). Our findings suggest that single-occupancy rooms promote better sleep, yet additional research using objective sleep assessments (e.g., actigraphy or polysomnography) is needed to comprehensively evaluate environmental influences on sleep quality.

Although length of hospitalization has been proposed as a factor influencing sleep, our analysis did not reveal a significant association (*p* > 0.05, η^2^ < 0.02). However, patients hospitalized for ≥16 days exhibited a trend toward poorer sleep quality, possibly due to long-term exposure to hospital-related disruptions, increased medical interventions, and prolonged psychological distress (52, 53).

### 4.5 Study limitations and future research directions

#### 4.5.1 This study has several limitations

(1) Cross-Sectional Design—The study’s cross-sectional nature limits causal inference. Future longitudinal studies should track changes over time to clarify the directionality of the relationship between negative emotions and sleep disturbances.
(2) Self-Reported Measures—A key limitation of this study is the reliance on self-reported sleep data, specifically using the Pittsburgh Sleep Quality Index (PSQI). While the PSQI is a validated tool, it is subject to recall bias and does not provide objective sleep parameters, such as sleep architecture or respiratory disturbances. To enhance data accuracy and provide a more comprehensive understanding of sleep disturbances in gastric cancer patients, future research should incorporate objective measures, such as actigraphy or polysomnography (PSG), which would allow for a more detailed assessment of sleep patterns and related disruptions.
(3) Lack of Intervention Testing—While this study highlights the link between psychological distress and sleep disturbances, it does not assess intervention efficacy. Future studies should evaluate structured interventions, including cognitive-behavioral therapy (CBT), pharmacological approaches, and hospital-based sleep improvement strategies.
(4) Potential Confounding Factors—Variables such as social support, treatment regimens, and hospitalization environment were not fully controlled. Future research should use multivariate analyses to adjust for these confounders and explore their impact on emotional well-being and sleep quality.
(5) Generalizability—As this study was conducted at a single medical center, findings may not be fully generalizable. Multi-center studies with larger, more diverse populations are needed to validate results.
(6) Comorbidities—Although patients with uncontrolled metabolic or cardiovascular conditions were excluded, controlled hypertension and diabetes were present in a subset of participants. Future studies should examine the potential impact of these comorbidities on sleep and emotional well-being.

By addressing these limitations, future research can provide stronger evidence for targeted interventions that improve psychological health and sleep quality in gastric cancer patients.

#### 4.5.2 Future research directions and interventions

To maximize clinical impact, future research should prioritize testing evidence-based interventions through rigorous study designs rather than solely proposing theoretical approaches. Key areas for future investigation include:

(1) Psychological Interventions.

Randomized controlled trials (RCTs) should evaluate the effectiveness of cognitive-behavioral therapy (CBT), mindfulness-based stress reduction (MBSR), and other psychological interventions in alleviating emotional distress and improving sleep quality in gastric cancer patients.

(2) Hospital-Based Environmental Modifications.

Research should investigate whether optimizing hospital environments, such as noise reduction protocols, single-room accommodations, and improved lighting conditions, can enhance sleep quality and overall well-being.

(3) Pharmacological and Integrative Approaches.

Comparative studies should assess the efficacy of melatonin, sedatives, anti-inflammatory agents, and other pharmacological treatments for managing sleep disturbances while minimizing adverse effects.

(4) Longitudinal Studies on Prognosis and Treatment Outcomes.

Prospective cohort studies should examine the long-term bidirectional relationship between negative emotions and sleep disturbances and its impact on cancer progression, treatment adherence, and overall survival rates.

(5) Digital Health and Real-Time Monitoring.

The integration of mobile health (mHealth) applications and wearable technology could facilitate continuous real-time monitoring of emotional distress and sleep patterns, enabling early detection and timely intervention.

(6) Non-Invasive Sleep Monitoring Technologies.

Future studies should incorporate objective sleep assessments using actigraphy or wearable sleep tracking devices to enhance data reliability while reducing patient burden compared to polysomnography.

By advancing research in these areas, future studies can contribute to the development of personalized, multidisciplinary intervention strategies, ultimately improving the mental health, sleep quality, and overall quality of life for gastric cancer patients.

### 4.6 Clinical implications

The findings of this study emphasize the need for a multidisciplinary approach to managing psychological distress and sleep disturbances in gastric cancer patients. Clinical recommendations include:

(1) Routine psychological screening to identify patients at risk for severe anxiety and depression.
(2) Personalized sleep management strategies, including environmental modifications, sleep hygiene education, and pharmacological treatments where appropriate.
(3) Strengthening social support networks, integrating family-based interventions into routine cancer care.
(4) Optimizing hospitalization conditions, such as reducing nighttime disturbances and improving ward design to promote better sleep.

By addressing these factors, future clinical strategies can enhance both mental well-being and sleep quality, ultimately improving patient outcomes and quality of life.

## 5 Conclusion

This study highlights the prevalence of negative emotions and sleep disturbances in gastric cancer patients, with a significant negative correlation between the two. Key factors such as gender, age, and tumor stage were found to influence emotional distress and sleep quality, with female patients, older individuals, and those in advanced stages experiencing greater challenges. The hospital environment, particularly single-occupancy rooms, also contributed to improved sleep quality.

Given the bidirectional relationship between negative emotions and sleep, integrated interventions addressing both psychological and sleep health are essential. Clinical strategies should include routine psychological screenings, personalized sleep management, and optimizing the hospital environment. Future research should focus on longitudinal studies and intervention evaluations to further understand and improve the care of gastric cancer patients.

In conclusion, addressing both emotional and sleep-related issues is crucial for improving the well-being and clinical outcomes of gastric cancer patients.

## Data Availability

The raw data supporting the conclusions of this article will be made available by the authors, without undue reservation.
